# On-chip background dilution in droplets with high particle recovery using acoustophoresis

**DOI:** 10.1063/1.5129256

**Published:** 2019-12-06

**Authors:** Zhenhua Liu, Anna Fornell, Laurent Barbe, Klas Hjort, Maria Tenje

**Affiliations:** 1 Department of Engineering Sciences, Uppsala University, 75271 Uppsala, Sweden; 2 Science for Life Laboratory, Uppsala University, 75271 Uppsala, Sweden

## Abstract

Droplet microfluidics has shown great potential for on-chip biological and chemical assays. However, fluid exchange in droplet microfluidics with high particle recovery is still a major bottleneck. Here, using acoustophoresis, we present for the first time a label-free method to achieve continuous background dilution in droplets containing cells with high sample recovery. The system comprises droplet generation, acoustic focusing, droplet splitting, picoinjection, and serpentine mixing on the same chip. The capacities of the picoinjection and the droplet split to dilute the background fluorescent signal in the droplets have been characterized. The sample recovery at different droplet split ratios has also been characterized. The results show a maximum of 4.3-fold background dilution with 87.7% particle recovery. We also demonstrated that the system can be used to dilute background fluorescent signal in droplets containing either polystyrene particles or endothelial cells.

## I. INTRODUCTION

Droplet microfluidics provides a platform for the generation of monodisperse droplets at high throughput and the technology has found many applications in biological sciences, from single cell RNA sequencing^1^ and single cell antibiotic resistance screening^2^ to single organism behavior studies.^3^ One of the main applications of the technology is to use the droplets as miniaturized reaction chambers for individual biological experiments and measurements. The volume of each droplet is typically in the picoliter to nanoliter range; thus, the consumption of sample and reagents are orders of magnitude lower compared to standard methods, reducing the cost for each analysis.

To enable the integration of complete biological assays into the droplet microfluidic format, different technical solutions to handle and analyze the droplets on-chip are needed. For example, unit operations have been developed to split,^4^ merge,^5^ sort,^6–8^ and inject fluid^9^ into droplets. To enable more complex processes, several unit operators need to be added in sequence on-chip. One such combination that would significantly lift the potential of the droplet microfluidics toolbox is to perform fluid exchange in droplets without the loss of the encapsulated particles. Such a unit operator combination could be used to wash particles encapsulated inside droplets, perform buffer exchange, or reduce background signal in droplets. Ideally, such droplet washing unit operation should be label-free, continuous, easy to integrate, and with the potential to be operated with different biological samples at high throughput.

Different technical solutions to wash beads and cells encapsulated inside droplets have previously been demonstrated in the literature. For example, Mary *et al.* presented a method to decrease the background fluorescent signal in droplets by first adding fresh medium to the original droplet and consecutively split the droplet in three succeeding T-junctions to return the droplet volume to its original value.^10^ The downside of this method is that the concentration of particles is also diluted, which results in a large number of empty droplets. This limitation can be solved by controlling the position of the particles during the droplet split, and the most common way to achieve droplet washing with high particle recovery is by magnetophoresis.^11–14^ For example, Pan *et al.*^11^ showed a system with two droplet injection units combined with three droplet splits and applied the system for DNA extraction, and Doonan *et al.*^14^ showed another type of on-chip droplet washing system also using magnetic forces. Magnetic methods are generally simple to operate but have a limitation that they only work with magnetic particles or with magnetically labeled samples. Park *et al*. presented a method not requiring magnetic beads where they used surface acoustic waves to achieve droplet washing by first pushing the encapsulated polystyrene particles to the end of the droplet, splitting the droplet in half, followed by merging with a second droplet and splitting in half again.^15^ However, in their work, they only showed washing of polystyrene particles and not cells. In this work, we present for the first time a system capable of background dilution in droplets containing cells with high recovery using bulk acoustic waves. Another advantage of our system is that it is easy to integrate into a complete microfluidic circuit as all the steps are performed continuously.

The microfluidic platform we have developed for exchanging fluid inside droplets is operated by first using bulk acoustic waves to control the position of the particles in a trifurcation droplet split followed by injecting new fluid to restore the original droplet volume. Fluid is injected into the droplets by the widely used picoinjection technique as it is simple to design and fabricate, and the injected volume can be controlled precisely.^9^ With our system integrated with acoustophoresis and picoinjection, we show a 4.3-fold background dilution with 87.7% particle recovery. Furthermore, we have demonstrated droplet washing using both encapsulated fluorescent beads and endothelial cells.

## II. ACOUSTOPHORESIS

Acoustophoresis is a common method to focus, enrich, and sort bioparticles in microfluidic channels.^16–23^ Acoustic particle manipulation can be achieved using different actuation methods such as bulk acoustic standing waves,^16,17^ traveling surface acoustic waves,^18^ standing surface acoustic waves,^19^ sharp edge based systems,^21^ and acoustic bubble based systems.^22^ Recently, acoustophoresis has been applied to focus and enrich cells and plastic microbeads encapsulated inside water-in-oil droplets using either bulk acoustic waves or traveling surface acoustic waves.^15,16,23^ In this work, we use bulk acoustic waves.

Commonly, bulk acoustic wave devices are operated by generating a half-wavelength acoustic standing wave field in the microchannel by an external piezoelectric transducer. To enable the build-up of a strong acoustic standing wave field, the device should be fabricated in a material with high acoustic impedance such as glass or silicon.^24^ At half-wavelength resonance, there will be a pressure node along the centerline of the microchannel and there will be pressure antinodes at the walls. Because of the acoustic standing wave field, particles will be pushed to specific locations in the microchannel by the primary acoustic radiation force. Particles with a positive acoustic contrast factor (Φ>0) are moved to the pressure node while particles with a negative acoustic contrast factor (Φ<0) are moved to the pressure antinodes. The primary acoustic radiation force, $F^{\mathrm{rad}}$, acting on a small particle in a half-wavelength standing wave field is given by^25^

$$F^{\mathrm{rad}}=4\pi \Phi (\tilde{\kappa },\tilde{\rho })ka^{3}E_{\mathrm{ac}}\;\sin(2ky),$$ (1a)

$$\Phi =\frac{1}{3}\left(\frac{5\tilde{\rho }-2}{2\tilde{\rho }+1}-\tilde{\kappa }\right),$$ (1b)

where *k* is the wavenumber (k=2π/λ and λ is the wavelength), *a* is the particle radius, $E_{\mathrm{ac}}$ is the acoustic energy density, *y* is the distance from the channel wall, $\tilde{\rho }$ is the density ratio between the particle and the fluid, and $\tilde{\kappa }$ is the compressibility ratio between the particle and the fluid. Most cells and plastic microbeads are denser and stiffer than water (Φ>0) and are hence moved to the pressure node.

## III. METHODS

### A. Chip design and fabrication

**FIG. 1.**
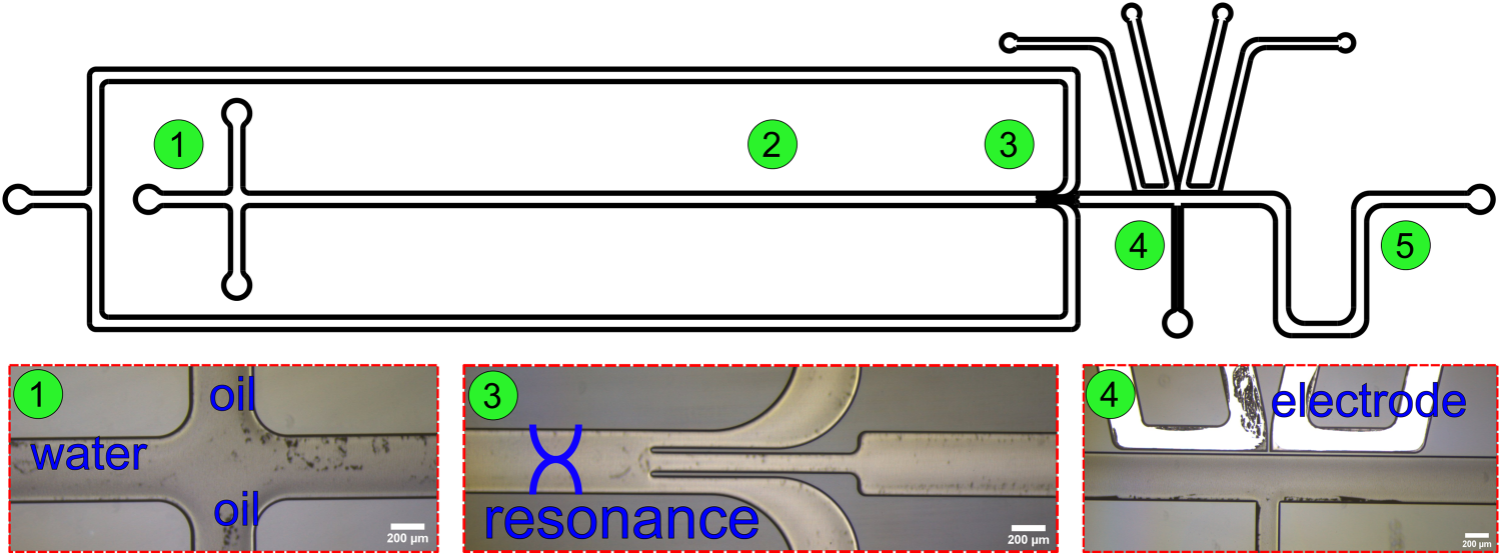
The design of the chip. The chip contains five different unit operators: (1) droplet generation, (2) acoustic focusing, (3) droplet split, (4) picoinjection, and (5) serpentine mixing.

The microfluidic chip contains five different unit operators: (1) droplet generation, (2) acoustic focusing, (3) droplet split, (4) picoinjection, and (5) serpentine mixing (Fig. 1). The acoustic focusing channel is 380 *μ*m wide, and the droplet split channels are 120 *μ*m wide each. The channel width of the picoinjector is 100 *μ*m, and the distance between the main microfluidic channel and the picoinjector electrodes is 20 *μ*m. The height of all channels is 100 *μ*m.

Droplets are generated in squeezing mode in a cross-flow channel at a rate between 0.5 Hz and 1.2 Hz. Both the dispersed phase and the continuous phase channels have separate inlets where the flow rate is controlled independently. As the droplets travel down the channel driven by the syringe pump, the encapsulated beads or cells are aligned in the center of the droplets by the acoustic radiation force. The droplets flow through the trifurcation channel where they are split into three daughter droplets with the particles remaining in the center daughter droplet due to the acoustic alignment. The two side split channels are joined to a common outlet channel, and the flow rate is controlled from a common syringe pump. The center daughter droplets then pass the picoinjector where clean water is injected to dilute the droplets. The serpentine channel enhances mixing of the injected fluid.

**FIG. 2.**
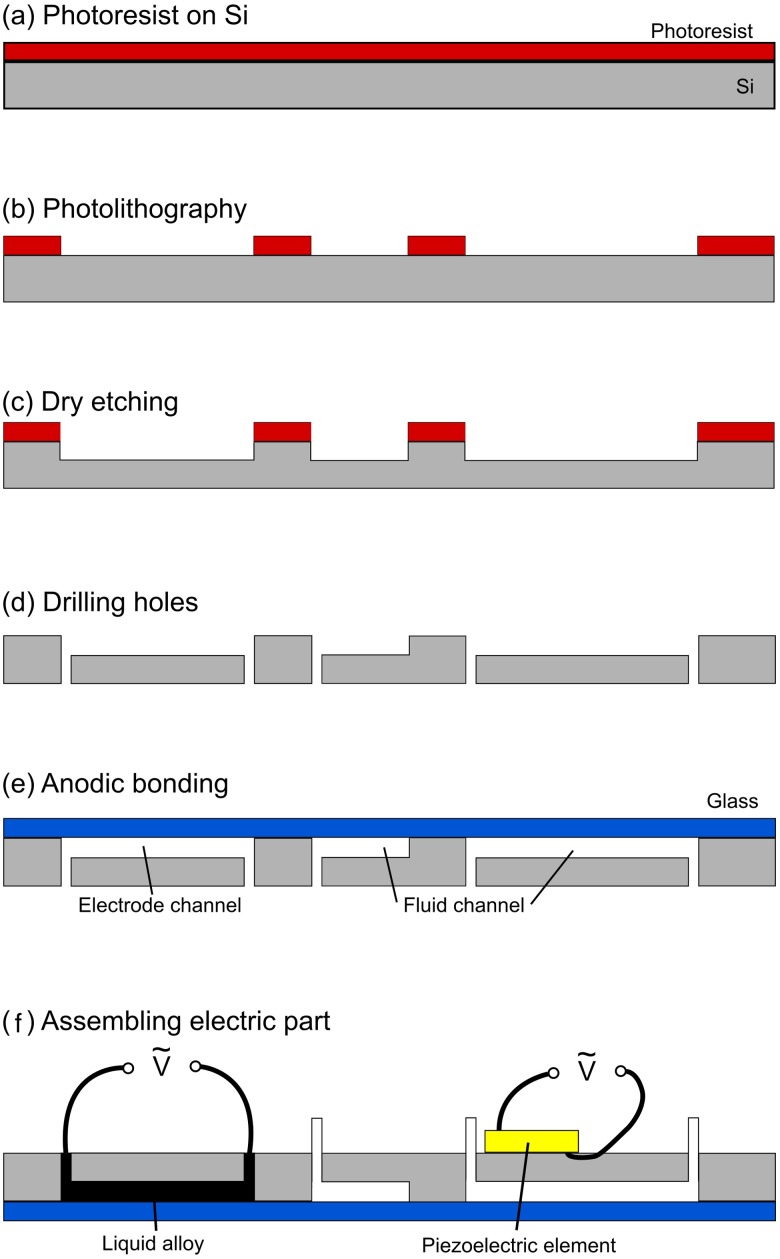
The fabrication process of the chip: (a) Photoresist was spin-coated on a high-resistivity silicon wafer, (b) the photoresist was patterned by UV lithography, (c) the microchannels were dry etched, (d) inlet and outlet holes were drilled, (e) the silicon wafer was sealed by anodic bonding with a glass wafer, and (f) the liquid alloy was injected into the electrode channels and a piezoelectric element was glued on the chip.

The microfluidic channels were etched using a standard dry etching process^7^ (Fig. 2) on a high-resistivity silicon wafer (resistivity ≥ 30 000 Ω cm, Topsil). After etching, the microfluidic chips were sealed by anodic bonding of a glass wafer and diced into individual chips. For the picoinjector electrodes, liquid alloy (Galinstan, Geratherm) was injected into the channels. A hydrophobic coating silane (Repel-silane, Sigma-Aldrich) was injected into the fluid channels prior to use. A piezoelectric element with optimal frequency of 2 MHz (APC-840, Americanpiezo) was glued onto the silicon chip using cyanoacrylate glue (420, Loctite). The electrodes for the picoinjector and the piezoelectric element were connected to a two-channel function generator (AFG3022C, Tektronix). The two signals for the picoinjector and the piezoelectric element were amplified by two separate power amplifiers (210L, Electronics & Innovation, and XPA125B, Xiegu). An oscilloscope (TBS 1102B, Tektronix) was used to monitor the applied signals.

### B. Analysis methods

Droplet images were captured using a camera with a CMOS sensor (DFK NME33UX174, IMAGINGSOURCE) mounted on a fluorescence microscope (TE2000-U, Nikon) at three different positions on the chip as indicated in Fig. 1: (3) just before the droplet split, (4) after the droplet split, and (5) after passing through the serpentine mixing channel following the picoinjector. As it was not possible to study individual droplets at all these three places simultaneously due to the limited visual field of the microscope, first images of 50 consecutive droplets at positions 3 and 4 were captured and then the microscope stage was moved to position 5 to record images of another 50 droplets. The average values of the 50 droplets at the three different positions were used for calculating the droplet volumes. The volume of droplets was calculated as^26^

$$V=\left[HW-(4-\pi ){\left(\frac{2}{H}+\frac{2}{W}\right)}^{-2}\right]\left(L-\frac{W}{3}\right),$$ (2)

where *H* is the height of the channel, *W* is the width of the channel, and *L* is the length of the droplet. The width and length of the droplets were determined by an image processing program (Fiji, ImageJ) from the threshold of the light intensity.

The initial concentration of the used solutions was known, so the concentration of the droplet at the end of the serpentine channel was calculated using the law of mass conservation,

$$C_{1}V_{1}=C_{2}V_{2},$$ (3)

where *C*_1_ and *V*_1_ are the background concentration and volume of the original droplet, and *C*_2_ and *V*_2_ are the background concentration and volume of the droplet after the washing step, respectively.

Fluorescent polystyrene beads (10 *μ*m, ThermoFisher) were added to the dispersed aqueous phase to evaluate the particle recovery. The visual field of the microscope was set at the split channel so that a video could be captured of 20 individual droplet splits at position 3. Still images displaying the same droplet right before and directly after the split were acquired, and the encapsulated beads were manually counted. The particle recovery of each droplet was determined as the number of beads in the center daughter droplet after the split divided by the number of beads in the droplet before the split. We evaluated the precision of the measurements using 20 and 50 droplets when the flow rates of the continuous phase, the dispersed phase, and the split side channels were 2 *μ*l min^−1^ for each inlet, 2 *μ*l min^−1^, and 4.5 *μ*l min^−1^, respectively. At a confidence level of 95%, the recovery of beads was 88.8 ± 6.8% for n = 50 and 87.5 ± 7.0% for n = 20 with acoustic actuation, and 28.9 ± 5.0% for n = 50 and 28.9 ± 5.0% for n = 20 without acoustic actuation. The F-ratio in ANOVA (analysis of variance) is 0.9 with acoustic actuation and 1.3 without acoustic actuation, which is less than the critical F value of 1.8 from the F distribution table (95% confidence level). Therefore, there is no significant difference in recovery of beads between sampling 20 or 50 droplets. Hence, to simplify the workload of counting the beads, 20 droplets were analyzed for all particle recovery experiments.

### C. On-chip droplet background dilution

Light mineral oil (Sigma-Aldrich) with 2% Span-80 (Sigma-Aldrich) was used as the continuous phase. The dispersed aqueous phase used was a fluorescent solution [1 mg ml^−1^ fluorescein sodium salt diluted in de-ionized (DI) water, Sigma-Aldrich] either with or without microbeads or cells. Liquid was injected into the system using syringe pumps (Nemesys, Cetoni). Plastic syringes (1 ml Syringe Luer-Lok Tip, Becton Dickinson) were used for the continuous phase, and glass syringes (1 ml L-Mark, Setonic) were used for the dispersed aqueous phase. The split ratio of the droplets was controlled by adjusting the withdrawal flow rate of the split side channels from a common syringe pump. Another glass syringe filled with clean water was connected to the picoinjector, and the fluid was injected using a syringe pump. At the end of the chip, the outlet was open to the air. All syringes were connected to the chip using polyethylene tubing (Intramedic, Becton Dickinson).

To demonstrate the droplet background dilution, fluorescent solution was used as the dispersed phase when measuring the volume of the droplets. Polystyrene beads were added to the dispersed phase to show the particle recovery capability. The ability of cells in droplet washing was demonstrated by encapsulation of endothelial cells (bEnd.3, ATCC) stained with a green cell tracker (10 *μ*M, DMEM Glutamax, Thermofisher). A solution of 2.3 × 10^7^ cells ml^−1^ and the background concentration of 2 *μ*M cell tracker were used for the experiments.

To generate the acoustic standing wave field in the channel, a sine wave was applied over the piezoelectric element with an amplitude of 23 V_pp_ and a frequency of 1.81 MHz. A square wave was applied on the electrodes for the picoinjection with an amplitude of 78 V_pp_ and a frequency of 5 kHz.

## IV. RESULTS

### A. Characterization of the picoinjector

**FIG. 3.**
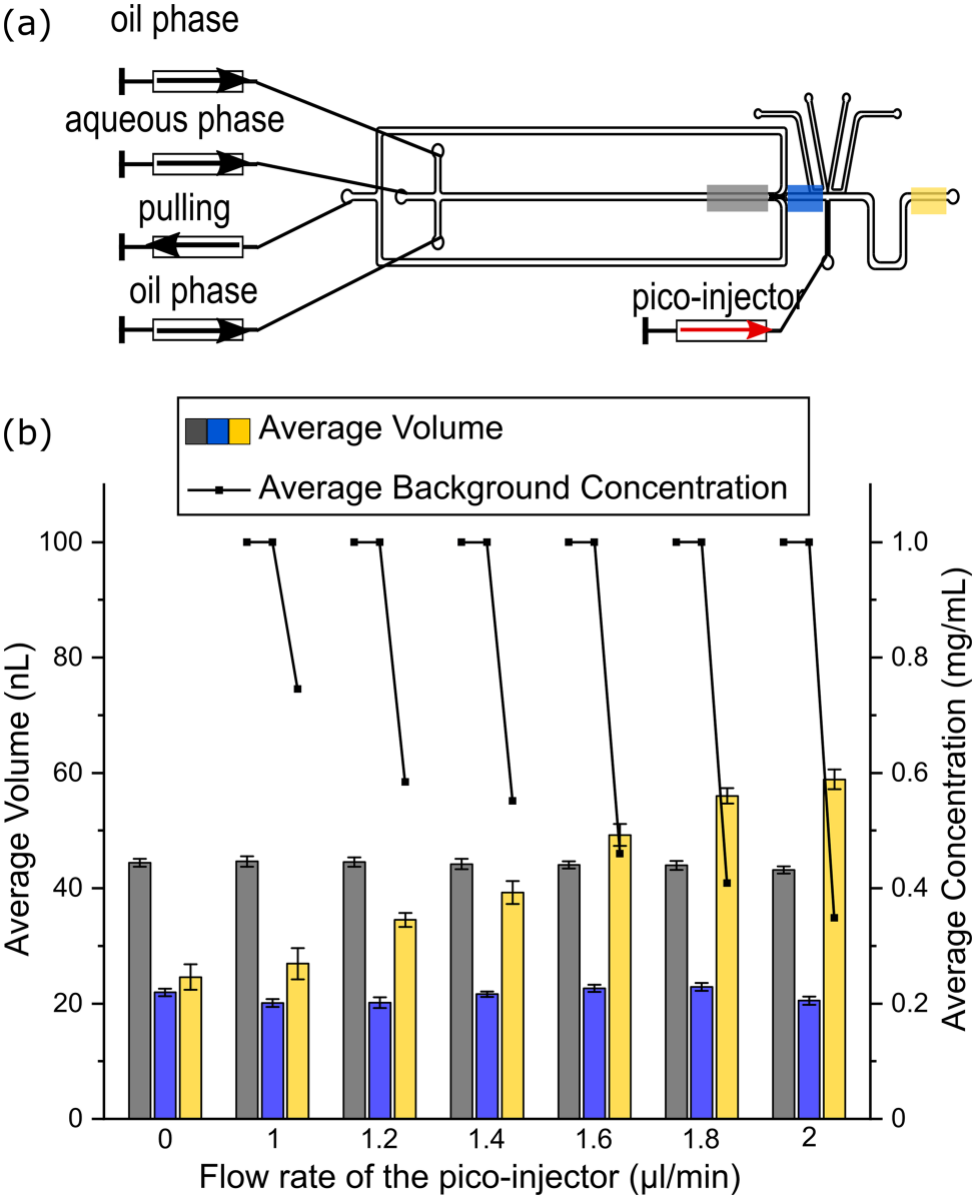
(a) The flow rate of the droplet generation and the droplet split was kept constant, and the flow rate of the picoinjector was varied. The volume and background concentration of the droplets were measured at position 3 (gray area), position 4 (blue area), and position 5 (yellow area). (b) The bars present the volumes of the original droplets (gray), the droplets after split (blue), and after the picoinjection (yellow). The concentrations of the fluorescent solutions at the same positions are represented by the dots. Error bars represent the standard deviation.

To characterize the capability of the picoinjector and identify its optimal flow rate, a set of experiments was performed where the injection flow rate of the picoinjector was systematically varied. The flow rate of the droplet generation and the droplet split was kept constant to ensure the same volume of the original droplets [Fig. 3(a)]. The flow rates of the dispersed phase, the continuous phase, and the total split channel outlet were 2 *μ*l min^−1^, 2 *μ*l min^−1^ for each inlet, and −3 *μ*l min^−1^, respectively. As shown in Fig. 3(b), when the electrodes for the picoinjection were active, the volume of the droplets after the picoinjection increased as the flow rate of the picoinjector was increased. The background concentration was decreased with an increase of the picoinjector flow rate, as expected from Eq. (3). The ideal flow settings of this picoinjection lay between 1 *μ*l min^−1^ and 2 *μ*l min^−1^. When the flow rate of the picoinjector was higher than 2 *μ*l min^−1^, satellite droplets were generated from it, which disturbed the analysis further downstream. The volume of the droplets before the droplet split (position 3) and after the droplet split (position 4) stayed constant at different flow rates of the picoinjector, which means the effect of back flow from the picoinjector can be neglected for droplet generation and droplet split.

### B. Characterization of the split ratio

When the droplet flows through the split channel, it is divided into three daughter droplets. The volume of each daughter droplet is determined by the ratio between the flow rates of the center and the split side channels and is controlled by the withdrawal flow rate of the split side channels. A higher flow rate of the split side channels results in smaller center daughter droplets after the split.

**FIG. 4.**
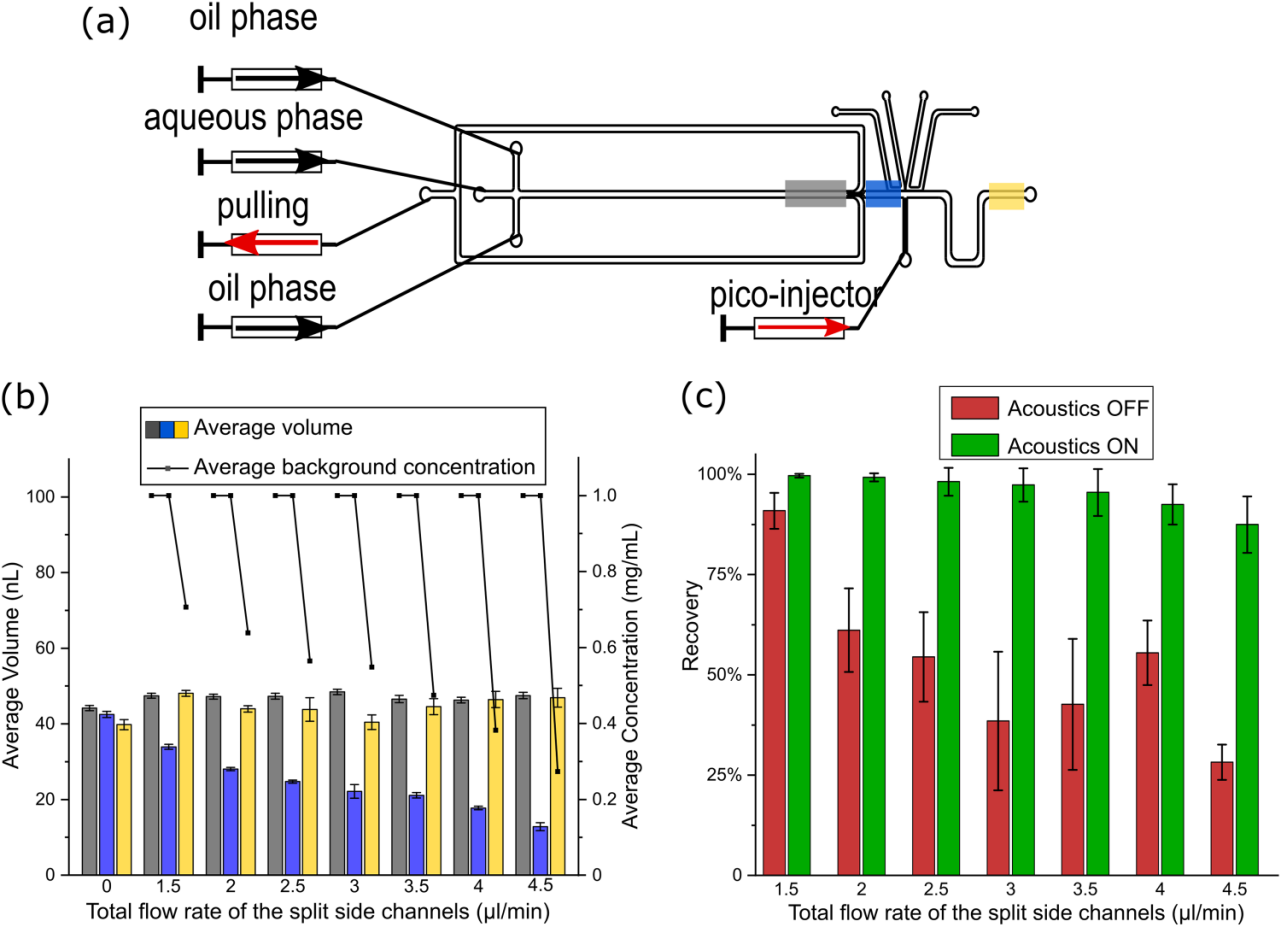
(a) The flow rate of the droplet generation was kept constant, and the flow rate of the pulling channel was varied to achieve different split ratios*.* The volume and background concentration of the droplets were measured at position 3 (gray area), position 4 (blue area), and position 5 (yellow area). (b) The bars present the volumes of the original droplets (gray), the droplets after the split (blue), and after the picoinjection (yellow). The concentrations of the fluorescent solutions at the same positions are represented by the dots. (c) The recovery of beads after the droplet split without (red) and with (green) acoustic actuation. Error bars represent the standard deviation.

To evaluate the effect of the split ratio on the final droplet background dilution, the flow rates of the split side channels were varied systematically. The flow rate of the continuous and the dispersed phase was kept constant (all at 2 *μ*l min^−1^) to ensure the same volume of the original droplets. After the split, the volume of the droplet was returned to its original volume by injecting different volumes of clean water with the picoinjector. The volume and concentration of the droplets were measured at positions 3 and 4, as well as after the picoinjector at position 5, as described above [Fig. 4(a)]. The recovery of beads in the droplets was measured at the droplet split (Videos 1 and 2 in the supplementary material).

When the flow rate of the split side channels and the picoinjector were both set at 0 *μ*l min^−1^, the volume of the droplets before and after the split as well as after the picoinjection were all the same [Fig. 4(b)]. As the total flow rate of the split side channels increased from 0 *μ*l min^−1^ to 4.5 *μ*l min^−1^, the volume of the center daughter droplet was decreased from 42.5 ± 0.8 nl to 12.8 ± 1.1 nl but could still be returned to its original volume by injection of liquid from the picoinjector. A larger flow rate of the split side channels resulted in higher droplet background dilution after the picoinjector, as expected from Eq. (3). However, when the total flow rate of the split side channels was higher than 4.5 *μ*l min^−1^, the center daughter droplets were so small that they coalesced with each other before the picoinjection, thus setting the maximum value of the flow rate of the outlet channels in this design.

In addition to the droplet background dilution, the particle recovery was characterized in relation to the flow rate ratios following the method described above. When the acoustics was off, the recovery of beads was reduced as the flow rate of the outlet channel increased, showing that the beads were randomly distributed in the daughter droplets. When the acoustic was on, the recovery of the beads reached ∼90% for all flow rates from 1.5 *μ*l min^−1^ to 3.5 *μ*l min^−1^. At the highest flow rate (4.5 *μ*l min^−1^), the recovery of beads was still 87.5% ± 7.0% with the acoustics activated, as compared to only 28.2% ± 4.4% without acoustic actuation [Fig. 4(c)].

### C. Background dilution in droplets

The results in the previous sections show the characterization of all individual parts of the droplet washing system and their respective effect on reaching maximum droplet background dilution with a high particle recovery. In the supplementary material, additional characterization data on the effect of the original droplet size and the total flow rate on the background dilution efficiency and the recovery of beads are presented. The results show the ability to dilute the background signal in droplets with high particle recovery for different original droplet sizes and different total flow rates.

To achieve maximum background dilution, the volume of the droplet after the split should be as small as possible and the volume of the droplet after picoinjection should be as large as possible. Based on the characterization above, the flow rate of the dispersed phase was set to 2.6 *μ*l min^−1^, and the flow rate of each inlet for the continuous phase was set to 1.7 *μ*l min^−1^ to generate droplets with an original volume of 51.9 ± 0.7 nl. To minimize the volume of the center daughter droplet at the split, the withdrawal flow rate of the split side channels was set to 4 *μ*l min^−1^, which was previously determined as the maximum flow rate with remained stability in the system. The average volume of the droplets after the split was decreased to 11.8 ± 0.7 nl. The flow rate of the picoinjector was set at 2 *μ*l min^−1^, which is the maximum flow rate for stable operation in this design. From optical images of the droplets at the end of the serpentine mixing channel (position 5), it could be measured and calculated that the background concentration in the droplets was decreased from 1.00 mg ml^−1^ to only 0.23 mg ml^−1^, representing a 4.3-fold background dilution with a bead recovery of 87.7% ± 4.3%.

### D. Background dilution in cell encapsulated droplets

**FIG. 5.**
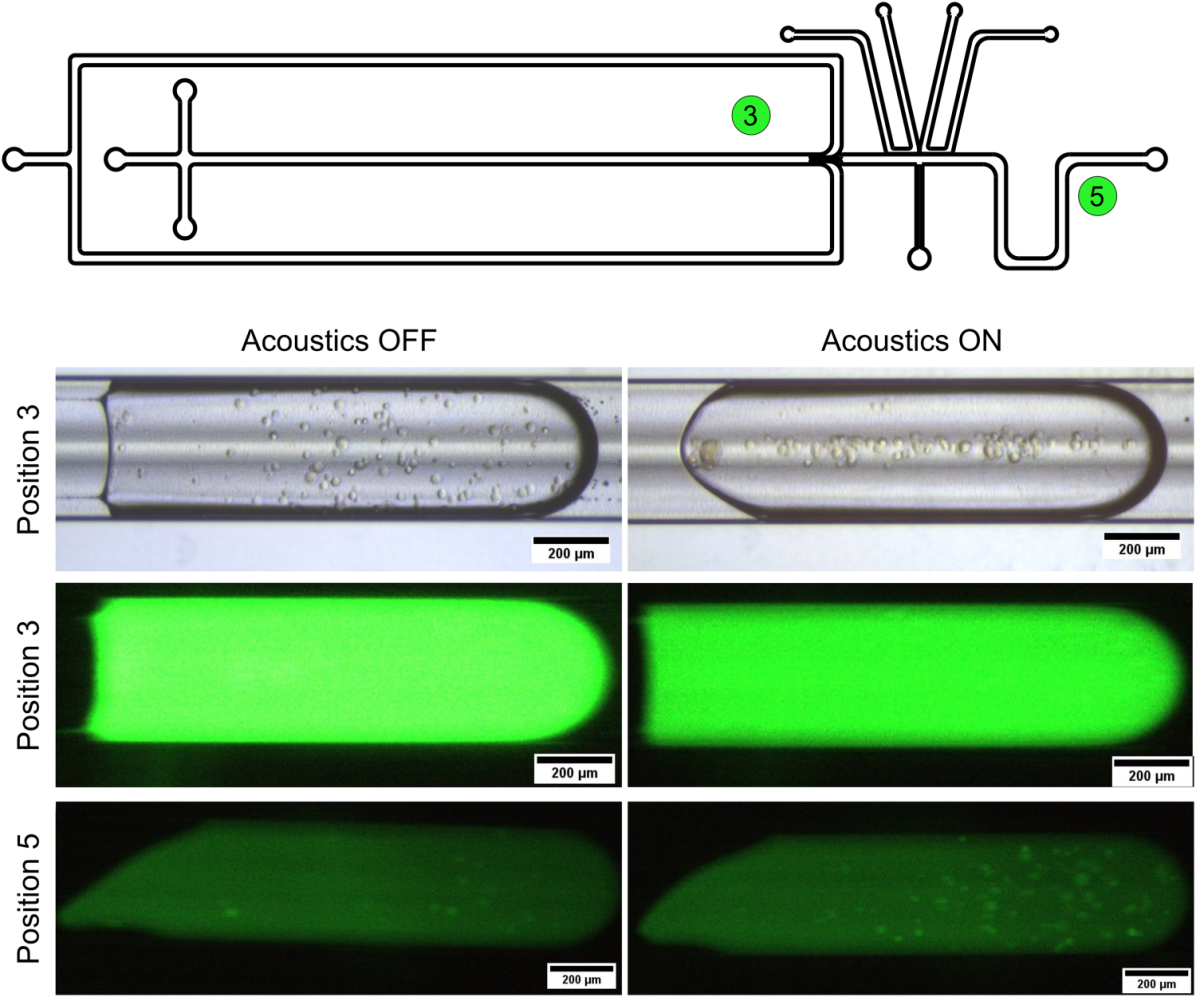
Optical images of droplets with encapsulated endothelial cells taken in bright field and fluorescent mode at two different positions in the microfluidic chip. In the original droplets, the cells were focused by the acoustics, but the high background noise hid the cells in the fluorescent images (at position 3). After the droplet washing, the background noise was diluted and the cells appeared in the fluorescent images and the cell recovery was higher when the acoustics was applied (at position 5). The serpentine mixing channel induces chaotic flow inside the droplets that the acoustic radiation force cannot compete with, which explains the lack of focusing observed for the cells in the bottom right image (at position 5).

To demonstrate the use of this new unit operator for on-chip background dilution with cells encapsulated in droplets, endothelial cells stained with a fluorescent cell tracker were used (Videos 3 and 4 in the supplementary material). The same flow rates as in the maximum background dilution experiment were used. Figure 5 shows optical images of the droplets with the encapsulated cells at positions 3 and 5 in the chip. In the bright field image, it can be seen that the cells were focused at the center of the droplet, but due to the high background fluorescent signal, the cells cannot be observed in the FITC (λ_EX _= 470–490 nm) channel of the microscope, although they had been labeled with a fluorescent stain. At position 5, the background noise was more than 4-fold diluted, and the cells could be observed in the FITC channel. The recovery of cells was high when the acoustic focusing was active, which was similar to what was demonstrated above using beads.

## V. CONCLUSIONS

We have designed and fabricated a microfluidic chip for on-chip background dilution of droplets with high particle recovery. This chip comprises five different unit operators that are integrated into a complete microfluidic circuit: droplet generation, acoustic focusing, droplet split, picoinjection, and serpentine mixing. We have characterized the capability of the picoinjection where the volume of the injected fluid can be controlled by the flow rate of the picoinjector. We have also characterized the droplet split by varying the withdrawal flow rate of the split side channels to minimize the volume of the daughter droplet. The main limitation for increasing the dilution efficiency in the current design is the droplet coalescence observed after the droplet split when the withdrawal flow rate of the split side channels exceeded 4.5 *μ*l min^−1^. This microfluidic system could achieve a maximum 4.3-fold background dilution (from 1.00 mg ml^−1^ to 0.23 mg ml^−1^) with a particle recovery of 87.7%. Moreover, we succeeded in applying this chip to performing background dilution in droplets with encapsulated cells at high cell recovery.

This microfluidic platform developed here has the ability to continuously dilute the background signal in droplets containing biological sample on-chip, and it should be straightforward to combine it with other droplet unit operators in the future.

## SUPPLEMENTARY MATERIAL

In the supplementary material, characterization data on the impact of the original droplet size and the total flow rate on background dilution and recovery are available, as well as videos showing acoustic focusing of beads and cells.
